# Ventricular conduction is a marker for arrhythmic risk in *SCN5A-*E1784K overlap sodium channel disease

**DOI:** 10.1093/europace/euag113

**Published:** 2026-05-11

**Authors:** Michael W T Tanck, Yanushi D Wijeyeratne, Julien Barc, Alison Muir, Takeshi Aiba, J Martijn Bos, Christian Veltmann, Joseph Galvin, Lia Crotti, Taisuke Ishikawa, Seiko Ohno, Stephen P Page, Isabelle Denjoy, David J Tester, Martina Muggenthaler, Kazuhiro Takahashi, Hariharan Raju, Jean Baptiste Gourraud, Richard Redon, Jean-Jacques Schott, Carla Haglund-Turnquist, Matteo Pedrazzini, Chiara Alberio, Federica Dagradi, Michael Papadakis, Sanjay Sharma, Edward Petzer, Dan M Roden, Minoru Horie, Peter J Schwartz, Naomasa Makita, Martin M Borggrefe, Michael J Ackerman, Wataru Shimizu, Pascal P McKeown, Vincent Probst, Elijah R Behr

**Affiliations:** Amsterdam UMC, University of Amsterdam, Epidemiology and Data Science, Amsterdam Public Health, Meibergdreef 9, Amsterdam, Netherlands; Cardiovascular and Genomics Research Institute, School of Health and Medical Sciences, City St George’s University of London, London SW17 0RE, UK; St George’s University Hospitals National Health Service (NHS) Foundation Trust, London, UK; Department of Medical Genetics, Nantes Université, CNRS, INSERM, L’institut du Thorax, Nantes F-44000, France; Belfast Health and Social Care Trust, Belfast, UK; Department of Cardiovascular Medicine, National Cerebral and Cardiovascular Center, Osaka, Japan; Departments of Cardiovascular Medicine (Division of Heart Rhythm Services), Pediatric and Adolescent Medicine (Division of Pediatric Cardiology), and Molecular Pharmacology & Experimental Therapeutics (Windland Smith Rice Sudden Death Genomics Laboratory), Mayo Clinic, Rochester, MN, USA; Center of Electrophysiology Bremen, Bremen, Germany; Mater Misericordiae University Hospital, Dublin, Ireland; Istituto Auxologico Italiano IRCCS, Center for Cardiac Arrhythmias of Genetic Origin and Laboratory of Cardiovascular Genetics, Milan, Italy; Department of Medicine and Surgery, University of Milano-Bicocca, Milan, Italy; Department of Cardiovascular Medicine, National Cerebral and Cardiovascular Center, Osaka, Japan; Department of Cardiovascular Medicine, National Cerebral and Cardiovascular Center, Osaka, Japan; Shiga University of Medical Science, Shiga, Japan; Leeds Teaching Hospitals NHS Trust, Leeds, UK; AP-HP Nord, Hôpital Bichat, Cardiology Department and Reference Center for Hereditary Arrhythmic Diseases, Paris Cité University, INSERM U1166, Paris, France; Departments of Cardiovascular Medicine (Division of Heart Rhythm Services), Pediatric and Adolescent Medicine (Division of Pediatric Cardiology), and Molecular Pharmacology & Experimental Therapeutics (Windland Smith Rice Sudden Death Genomics Laboratory), Mayo Clinic, Rochester, MN, USA; Cardiovascular and Genomics Research Institute, School of Health and Medical Sciences, City St George’s University of London, London SW17 0RE, UK; St George’s University Hospitals National Health Service (NHS) Foundation Trust, London, UK; Royal Devon University Healthcare NHS Foundation Trust, Exeter, UK; Kizawa Memorial Hospital, Gifu, Japan; Cardiovascular and Genomics Research Institute, School of Health and Medical Sciences, City St George’s University of London, London SW17 0RE, UK; St George’s University Hospitals National Health Service (NHS) Foundation Trust, London, UK; Faculty of Medicine, Health and Human Sciences, Macquarie University, Sydney, Australia; Nantes Université, CHU Nantes, CNRS, INSERM, L’institut du Thorax, Nantes F-44000, France; Nantes Université, CHU Nantes, CNRS, INSERM, L’institut du Thorax, Nantes F-44000, France; Nantes Université, CHU Nantes, CNRS, INSERM, L’institut du Thorax, Nantes F-44000, France; Departments of Cardiovascular Medicine (Division of Heart Rhythm Services), Pediatric and Adolescent Medicine (Division of Pediatric Cardiology), and Molecular Pharmacology & Experimental Therapeutics (Windland Smith Rice Sudden Death Genomics Laboratory), Mayo Clinic, Rochester, MN, USA; Istituto Auxologico Italiano IRCCS, Center for Cardiac Arrhythmias of Genetic Origin and Laboratory of Cardiovascular Genetics, Milan, Italy; Istituto Auxologico Italiano IRCCS, Center for Cardiac Arrhythmias of Genetic Origin and Laboratory of Cardiovascular Genetics, Milan, Italy; Istituto Auxologico Italiano IRCCS, Center for Cardiac Arrhythmias of Genetic Origin and Laboratory of Cardiovascular Genetics, Milan, Italy; Cardiovascular and Genomics Research Institute, School of Health and Medical Sciences, City St George’s University of London, London SW17 0RE, UK; St George’s University Hospitals National Health Service (NHS) Foundation Trust, London, UK; Cardiovascular and Genomics Research Institute, School of Health and Medical Sciences, City St George’s University of London, London SW17 0RE, UK; St George’s University Hospitals National Health Service (NHS) Foundation Trust, London, UK; Darent Valley Hospital, Dartford and Gravesham NHS Trust, Dartford, UK; Vanderbilt University School of Medicine, Nashville, USA; Shiga University of Medical Science, Shiga, Japan; Istituto Auxologico Italiano IRCCS, Center for Cardiac Arrhythmias of Genetic Origin and Laboratory of Cardiovascular Genetics, Milan, Italy; Department of Cardiovascular Medicine, National Cerebral and Cardiovascular Center, Osaka, Japan; Sapporo Teishinkai Hospital, Sapporo, Japan; Department of Medicine, University Medical Centre Mannheim (UMM), Faculty of Medicine Mannheim, University of Heidelberg, European Center for AngioScience (ECAS), and DZHK (German Center for Cardiovascular Research) Partner Site Heidelberg/Mannheim, Mannheim, Germany; Departments of Cardiovascular Medicine (Division of Heart Rhythm Services), Pediatric and Adolescent Medicine (Division of Pediatric Cardiology), and Molecular Pharmacology & Experimental Therapeutics (Windland Smith Rice Sudden Death Genomics Laboratory), Mayo Clinic, Rochester, MN, USA; Department of Cardiovascular Medicine, National Cerebral and Cardiovascular Center, Osaka, Japan; Department of Cardiovascular Medicine, Nippon Medical School, Tokyo, Japan; Belfast Health and Social Care Trust, Belfast, UK; Queen’s University Belfast, Belfast, UK; Nantes Université, CHU Nantes, CNRS, INSERM, L’institut du Thorax, Nantes F-44000, France; Cardiovascular and Genomics Research Institute, School of Health and Medical Sciences, City St George’s University of London, London SW17 0RE, UK; St George’s University Hospitals National Health Service (NHS) Foundation Trust, London, UK

**Keywords:** penetrance, Phenotype, ECG, Brugada syndrome, Long QT syndrome

## Abstract

**Aims:**

*SCN5A*-E1784K (c.5350G>A) is the most common variant associated with the long QT (LQTS) and Brugada syndromes (BrS). It can manifest variably as LQTS, BrS, and/or conduction disorders. This presents a challenge for risk stratification. We aimed to describe clinical and ECG characteristics and identify risk markers that associate with arrhythmic events.

**Methods and results:**

We undertook a retrospective observational multicentre study of a large cohort of 231 subjects with *SCN5A*-E1784K from Europe, USA, and Japan. Comprehensive demographic and clinical data, including initial presentation ECG and follow-up, were collected. ‘Lethal events’ were defined as sudden death, non-fatal cardiac arrest, and documented sustained VT or VF. ‘Cardiac events’ were defined as arrhythmic syncope plus any lethal events. Clinical characteristics and ECG parameters corrected for age were investigated for association with lethal and/or cardiac events. Fourteen (6%) subjects experienced a lethal event and 45 (19%) a cardiac event. PR interval and QRS duration were associated with lethal and cardiac events. In multivariable models, both PR interval and QRS duration were associated with lethal events, but only QRS duration was associated with cardiac events. Only age-corrected QRS (rQRS) was associated with lethal and cardiac event-free survival from birth after correction for multiple testing.

**Conclusion:**

Ventricular myocardial conduction appears likely to play a role in the risk of arrhythmic events in patients with *SCN5A*-E1784K. This provides an important opportunity for the personalization of management and has the potential to guide preventative therapies.

## Graphical Abstract

**Graphical Abstract:**
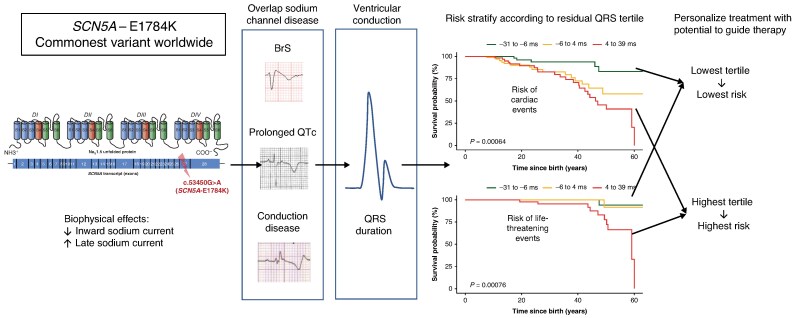

What’s new?

- QRS duration is associated with the risk of syncope and life-threatening arrhythmia in patients with *SCN5A*-E1784K. *SCN5A*-E1784K patients in the second and third QRS tertiles have a three- to five-fold increased risk of arrhythmic events.
- QTc did not associate with the risk of arrhythmic events in patients with *SCN5A*-E1784K.
- Ventricular myocardial conduction appears to play a role in the risk of life-threatening arrhythmic events in patients with overlap sodium channel disease, highlighting a potential target for future risk stratification and therapeutic guidance.

## Introduction

The *SCN5A* p.E1784K variant (glutamine to lysine substitution at position 1784; c.5350G>A; ClinVar ID: 9377) is a missense variant associated with an overlap syndrome that can manifest as long QT syndrome (LQTS), Brugada syndrome (BrS), and/or cardiac conduction disease (CCD) with variable severity. It is the most common pathogenic *SCN5A* variant worldwide accounting for a third of unrelated cases of the LQT3 subtype of long QT syndrome (LQTS) and 3% of Brugada syndrome (BrS).^1,2^ Affected individuals are at risk of potentially fatal ventricular arrhythmias, including torsades de pointes and ventricular fibrillation (VF). However, disease expression in *SCN5A*-E1784K patients is unpredictable, and features that determine arrhythmic risk remain incompletely defined. This presents a clinical challenge for risk stratification especially when mixed phenotypes increase the potential for drug interactions.^3^

*SCN5A*-E1784K exhibits a negative shift of steady-state sodium channel inactivation, resulting in a diminished inward sodium current, together with destabilized fast channel inactivation leading to a persistence of the late inward sodium current.^4,5^ These biophysical properties explain a mixed phenotype but not individual phenotypic variation with incomplete penetrance and variable expression of clinical phenotypes of LQTS, BrS, and/or CCD, even amongst affected relatives from the same pedigree.^4^

Previously, we have shown that BrS phenotype in *SCN5A-*E1784K is in part mediated by common genetic variation in a large international cohort of *SCN5A*-E1784K subjects.^6^ This well-characterized group represents the world’s largest collection of patients with *SCN5A*-E1784K and can be studied for clinical markers associated with arrhythmic risk. In the present manuscript, we describe the clinical characteristics observed in this *SCN5A*-E1784K cohort and aim to identify demographic and/or ECG markers associated with arrhythmic events and event-free survival.

## Methods

### Recruitment and case selection

Study participants were probands and relatives from families with *SCN5A*-E1784K under clinical care at 20 participating cardio-genetic centres in the UK, Ireland, France, Germany, Italy, USA, and Japan (see Supplementary material). IRB approval was obtained, according to the guidelines noted in Instructions to Authors. The study was approved by the West London Research Ethics Committee and the St George’s University of London and St George’s Hospital NHS Trust Joint Research and Enterprise Office. Local research ethics committee approval was obtained for participants recruited from other sites. Informed consent was obtained from all study participants.

### Clinical characteristics

Comprehensive clinical data were collected, including initial presentation and follow-up data, presenting (i.e. first recorded) ECGs, drug provocation tests, and pedigree data. ECG data included heart rhythm, the presence of the type 1 Brugada pattern, RR, PR, QRS, QRS angle, and QTc (Bazett’s formula). Two independent experienced observers made systematic manual measurements of presenting ECGs using electronic callipers (Cardio Calipers).^7^ QRS and QTc were measured in lead II or V5, wherever the end of the QRS or T wave was clearest. Where there was >5% discrepancy, measurements were repeated and re-compared. If a discrepancy remained, a third observer made measurements and a mean was taken. First ECGs were recorded prior to any medication being initiated.

### Definitions applied

BrS phenotype was defined as the presence of either a spontaneous or drug-induced type 1 Brugada pattern in at least one right precordial ECG lead in the standard or higher intercostal spaces.^8^ Intravenous drug provocation tests (ajmaline, flecainide, or pilsicainide) were performed as per local protocol. LQTS was defined as a QTc >470 ms for females and >450 ms for males. Normal QRS axis was defined as −30 to +90°. CCD was defined as presence of leftward axis deviation (QRS axis less than −30°) and/or QRS >120 ms, and/or the presence of any degree of atrioventricular block. ‘Lethal events’ were defined as sudden death, non-fatal cardiac arrest, or documented sustained VT or VF. ‘Cardiac events’ included sudden death, non-fatal cardiac arrest, documented sustained VT or VF, or arrhythmic syncope. Arrhythmic syncope was defined as a clinical diagnosis that excluded vasovagal characteristics and supported an arrhythmic origin as determined by the local cardiologist. Any cardiac events during an electrophysiological study or drug provocation test were excluded. Medications recorded were limited to antiarrhythmic drugs.

### Statistical analyses

Categorical variables were described as count and percentage and numerical variables were described as mean ± standard deviation (SD) or median and interquartile range (IQR), where appropriate. Normality of the numerical variables was checked using histograms and the Shapiro–Wilk test. Values between ancestries were compared using generalized linear mixed model with a kinship matrix to correct for related observations.

The effect of age at the time of ECG acquisition on ECG parameters was modelled using piecewise linear regression with a single breakpoint estimated using the observed data across the whole cohort. The possibility of sex-specific breakpoints and slopes was examined by inclusion of interactions terms, but no significant interactions were found. Subsequently, the residuals, i.e. the difference between observed value and the predicted age-corrected value, were calculated for each individual. These age-corrected ECG variables are subsequently referred to as residual PR (rPR), residual QRS (rQRS), residual QTc (rQTc) and residual RR (rRR). (NB. Restricted cubic spline models with 3 to 5 knots did not outperform the piecewise linear regression models in the present study.) For QRS duration, *SCN5A*-E1784K subjects were also classified using more conventional QRS categories: <100, 100–120, and >120 ms.

Associations between demographic and/or ECG markers with the occurrence of lethal (primary outcome) and/or cardiac (secondary outcome) events were tested using logistic regression with generalized estimation equations (GEE) to correct for related observations. In case of multiple significant variables, an additional multivariable logistic regression analysis was performed with all significant predictors with correction for ancestry and relatedness.

Kaplan–Meier curves were calculated to show lethal or cardiac event free survival from birth in the *SCN5A*-E1784K subjects. Follow-up started at birth and ended at the date of the lethal or first cardiac event or, in case of no event, at the time of the latest medical review. Event-free survival was then stratified by tertiles of rQRS.

Due to the retrospective nature of the current research, there is a risk of bias (see Limitations section). Therefore, certain choices with respect to the endpoints and the main analysis were made. Although the number of lethal events was smaller than the number of cardiac events, the severity of these events reduced the likelihood of misattribution of outcome. In addition, the main analyses focused on symptomatic vs. asymptomatic instead of a possible more informative time to symptoms analysis. By selecting lethal events as our primary outcome and symptomatic yes/no as primary analysis, we mitigate some of the biases.

All analyses were carried out using R (version 4.0.2) software. *P*-values <0.05 were considered nominally statistically significant with correction for multiple testing then applied. Further statistical methods and additional sensitivity analyses can be found in Supplementary materials.

## Results

### Cohort demographics

Ancestry, clinical, and ECG characteristics of the *SCN5A*-E1784K families are shown in Supplementary material online, *Table S1*. The total cohort consisted of 335 subjects with 231 *SCN5A*-E1784K subjects and 104 *SCN5A*-E1784K negative relatives. Drug provocation testing data for BrS were only available for 120 (35.7%).

### *SCN5A*-E1784K subjects

**Figure 1:**
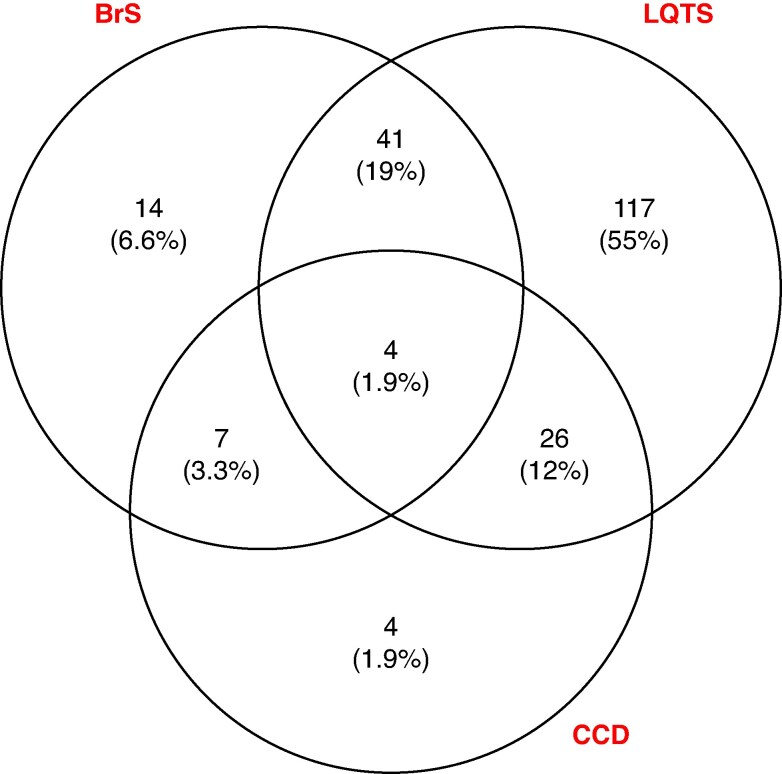
Venn diagram showing disease phenotypes in *n* = 231 *SCN5A*-E1784K subjects. Disease phenotype information was missing from 18 individuals (i.e. missing information on QTc, BrS status, and/or QRS). BrS, Brugada syndrome; LQTS, long QT syndrome; CCD, cardiac conduction disease.

The clinical and ECG characteristics of the 231 subjects with *SCN5A*-E1784K are shown in *Table 1*. Forty-seven per cent were male with a median age at recruitment of 25.9 years (IQR 29.3). Median family size was 2 (range 1–25) relatives with *SCN5A*-E1784K. Four pedigrees had >10 *SCN5A*-E1784K individuals, three European and one mixed ancestry. Different ancestry sub-groups were similar for sex, age at baseline and last follow-up, medication use (beta blocker and mexiletine), QRS duration, QRS axis, and BrS phenotype. Pacemaker and ICD implantation were higher in Europeans. The Venn diagram (*Figure 1*) presents disease phenotypes with the majority having presented with LQTS, mostly in isolation [*n* = 117 (55%)]. Japanese subjects had more LQTS phenotype and longer QTc and RR values. Drug provocation testing data for BrS were only available for 90 (39%) *SCN5A*-E1784K subjects.

**Table 1:** Clinical and ECG characteristics of *SCN5A*-E1784K at the time of the ECG recording. Differences between ancestries were analysed using generalized linear mixed models with a kinship matrix to correct for related observations. Statistically significant values (*P* < 0.05) are highlighted

	*SCN5A*-E1784K (*n* = 231)	European (*n* = 126)	Japanese (*n* = 65)	Other/mixed (*n* = 40)	*P*-value
Male	110 (47%)	59 (47%)	33 (51%)	18 (45%)	0.803
Age at baseline ECG (years)	25.9 (30.0)	26.3 (29.6)	17.1 (29.6)	31.7 (26.2)	0.381
Age at last follow up (years)	27.5 (30.1)	28.0 (30.0)	20.1 (29.3)	31.7 (26.2)	0.267
Medication	57 (25%)	41 (32%)	9 (14%)	7 (18%)	0.365
Beta-blocker (% of medication users)	47 (82%)	37 (90%)	3 (33%)	7 (100%)	0.961
Mexiletine (% of medication users)	22 (39%)	15 (37%)	7 (78%)	0 (0%)	0.799
Pacemaker	19 (8%)	14 (11%)	5 (8%)	0 (0%)	<0.001
ICD	37 (16%)	29 (23%)	5 (8%)	3 (8%)	0.001
BrS phenotype^c^	66 (29%)	46 (36%)	6 (9%)	14 (35%)	0.776
ECG parameters^a^					
PR interval (ms)	165 ± 30	163 ± 31	166 ± 27	170 ± 27	0.531
QRS duration (ms)	97 ± 13	97 ± 14	96 ± 12	95 ± 14	0.869
QRS axis (degrees)	61 (53)	57 (49)	70 (49)	53 (54)	0.138
Abnormal QRS axis	39 (17%)	20 (16%)	12 (18%)	8 (21%)	0.685
QTc (ms)	489 ± 31	478 ± 25	512 ± 28	487 ± 30	<0.001
LQTS^b^	188 (83%)	93 (76%)	64 (98%)	31 (79%)	0.012
RR interval (s)	0.92 ± 0.21	0.89 ± 0.21	1.01 ± 0.20	0.89 ± 0.20	0.001

Data are presented as count (%), mean ± SD or median (IQR)

^a^Available for *n* = 225, unless indicated otherwise

^b^LQTS was defined as a QTc >470 ms for females and >450 ms for males

^c^Drug challenge information only available for 90 individuals

### Therapies

Twenty-five per cent (57/231) received either beta-blockers and/or mexiletine during follow-up mainly for the treatment of LQTS: 86.7% (39/47) of subjects on beta-blockers and 90.1% (20/22) of subjects on mexiletine. An ICD was implanted in 37 (16.0%) of all 231 subjects, of whom 70.3% (26/37) had LQTS and 48.6% (18/37) had BrS, 10/37 (27%) having a mixed LQTS/BrS phenotype. A permanent pacemaker was implanted in a further 5.2% (12/231) of subjects.

#### ECG characteristics and age correction

The optimal break points for PR interval and QRS duration were 13 and 14 years, respectively, and 7 years for both QTc and RR (see Supplementary material online, material). All four ECG traits showed a larger increase per year before the break point (see Supplementary material online, *Table S2*, Supplementary material online, *Figure S1*). ECG characteristics for females and males are shown in Supplementary material online, *Table S3*.

#### Symptoms

Event status was available for all 231 subjects. There was no association between sex, disease phenotype, BrS phenotype or ancestry, and lethal or cardiac events.

Fourteen (6%) experienced a lethal event (*Table 2*). None were on beta-blocker or mexiletine at the time. Incomplete data were available on the circumstances of the event: one occurred during fever, five during rest, and eight were not documented. Subjects with lethal events were, on average, 26 years older at first ECG recording. PR, rPR, QRS, and rQRS were significantly prolonged (*Table 2*, Supplementary material online, *Table S4*). In a multiple logistic regression model with rPR and rQRS, the odds ratios were 1.03 [95%CI: 1.00–1.05, *P* = 0.027] and 1.08 [95%CI: 1.04–1.12, *P* = 0.0001] per ms deviation from the mean, respectively. Subjects in QRS categories 100–120 and >120 ms had significant higher odds for lethal events compared to QRS <100 ms (*Table 2*).

**Table 2:** Characteristics of *SCN5A*-E1784K per lethal and cardiac event category

	Lethal events				Cardiac events			
	No event (*n* = 217)	Event (*n* = 14)	OR [95% CI]	*P*-value	No event (*n* = 186)	Event (*n* = 45)	OR [95% CI]	*P*-value
Male	102 (47%)	8 (57%)	1.62 [0.57–4.62]	0.366	88 (47%)	22 (49%)	1.07 [0.60–1.90]	0.826
Age at baseline ECG (years)	23.6 (28.6)	49.3 (6.6)		0.007	22.4 (28.7)	39.7 (25.5)		0.003
≤20 years	95 (44%)	1 (8%)	REF		87 (47%)	9 (20%)	REF	
20–40 years	62 (29%)	10 (77%)	14.8 [1.80–121]	0.012	50 (27%)	22 (50%)	2.78 [1.15–6.68]	0.023
>40 years	58 (27%)	2 (15%)	3.16 [0.37–26.8]	0.292	47 (26%)	13 (30%)	4.45 [1.62–12.2]	0.004
Ancestry				0.693				0.693
European	116 (53%)	10 (71%)	REF		104 (56%)	22 (49%)	REF	
Japanese	63 (29%)	2 (14%)	0.45 [0.08–2.43]	0.354	50 (27%)	15 (33%)	1.42 [0.66–3.03]	0.371
Other/mixed	38 (18%)	2 (14%)	0.66 [0.21–2.08]	0.475	32 (17%)	8 (18%)	1.18 [0.59–2.36]	0.638
Disease phenotype				0.100				0.405
LQTS only	113 (52%)	4 (29%)	REF		96 (52%)	21 (47%)	REF	
BrS only	13 (6%)	1 (7%)	2.94 [0.47–18.5]	0.251	11 (6%)	3 (7%)	1.27 [0.34–4.71]	0.717
CCD only	3 (1%)	1 (7%)	11.6 [1.05–129]	0.045	2 (1%)	2 (8%)	4.02 [0.50–32.4]	0.192
BrS phenotype	61 (28%)	5 (36%)	1.42 [0.46–4.41]	0.543	55 (30%)	11 (24%)	0.77 [0.38–1.57]	0.472
ECG parameters^a^	(*n* = 213)	(*n* = 12)			(*n* = 182)	(*n* = 43)		
PR interval (ms)	163 ± 27	202 ± 42	1.04 [1.01–1.07]	0.010	162 ± 28	180 ± 32	**1.02 [1.01–1.03]**	**0.001**
Residual PR (rPR) (ms^b^)	−1 ± 23	25 ± 37	1.04 [1.01–1.06]	0.011	−2 ± 23	8 ± 30	1.01 [1.00–1.03]	0.093
QRS duration (ms)	96 ± 13	113 ± 14	**1.09 [1.06–1.13]**	**<0.001**	94 ± 12	106 ± 14	**1.07 [1.04–1.09]**	**<0.001**
Residual QRS (rQRS) (ms^b^)	−1 ± 12	15 ± 14	**1.09 [1.05–1.13]**	**<0.001**	−2 ± 12	7 ± 13	**1.06 [1.03–1.09]**	**<0.001**
QRS category^c^				0.003				**<0.001**
QRS <100 ms (*n* = 147)	145 (68%)	2 (17%)	REF		130 (71%)	17 (39%)	REF	
QRS 100–120 ms (*n* = 65)	58 (27%)	7 (58%)	7.2 [1.4–34.4]	0.014	47 (26%)	18 (42%)	2.4 [1.2–4.9]	0.013
QRS >120 ms (*n* = 14)	11 (5%)	3 (25%)	**14.3 [2.8–72.5]**	**0.001**	6 (3%)	8 (19%)	**8.5 [2.1–33.9]**	**0.003**
QRS axis (degrees)	63 (53)	44 (44)	0.99 [0.98–1.01]	0.230	61 (51)	56 (56)	1.00 [0.99–1.01]	0.485
Abnormal QRS axis	37 (17%)	2 (17%)	0.98 [0.21–4.53]	0.979	29 (16%)	11 (25%)	1.54 [0.71–3.36]	0.275
QTc (ms)	490 ± 31	484 ± 31	1.00 [0.97–1.03]	0.975	489 ± 31	492 ± 31	1.00 [0.99–1.01]	0.562
Residual QTc (rQTc) (ms^b^)	0 ± 29	−1 ± 31	1.00 [0.98–1.03]	0.508	−1 ± 29	3 ± 30	1.00 [0.99–1.02]	0.439
LQTS^d^	179 (84%)	9 (75%)	0.80 [0.14–4.52]	0.801	154 (84%)	35 (80%)	0.70 [0.32–1.54]	0.378
RR interval (s)	0.92 ± 0.21	1.03 ± 0.23	14.3 [0.66–307]	0.090	0.91 ± 0.21	0.98 ± 0.20	4.63 [0.84–25.6]	0.079
Residual RR (rRR) (s^b^)	0.00 ± 0.18	0.07 ± 0.22	8.05 [0.24–269]	0.244	−0.01 ± 0.18	0.03 ± 0.20	2.19 [0.29–16.5]	0.448

Data are presented as count (%), mean ± SD or median (IQR). Associations in bold passed the Bonferroni adjusted significance threshold (*P* < 0.003)

^a^Available for *n* = 225, unless indicated otherwise

^b^From mean of age group

^c^With adjustment for age at ECG in regression model (QRS missing from two individuals with lethal/cardiac event)

^d^LQTS was defined as a QTc >470 ms for females and >450 ms for males.

Forty-five subjects (19%) experienced a first cardiac event (*Table 2*). Subjects with an event prior to and during follow-up were significantly older (average 17 years) at first ECG recording. Two (4.4%) of the 45 first cardiac events were observed in patients receiving medication at the time. ECG data were available for 225 individuals (43 cardiac events, 12 lethal events). Subjects with a cardiac event had significantly longer PR, QRS, and rQRS values than those without (*Table 2*, Supplementary material online, *Table S4*). In a multiple logistic regression model with rPR and rQRS corrected for ancestry, the odds ratio for rPR and rQRS per ms deviation from mean were 1.01 [95%CI: 0.99–1.02, *P* = 0.301] and 1.05 [95%CI: 1.03–1.09, *P* = 0.0002], respectively. Subjects in QRS categories 100–120 and >120 ms had significant higher odds for cardiac events compared to QRS <100 ms (*Table 2*).

#### Event-free survival

**Figure 2:**
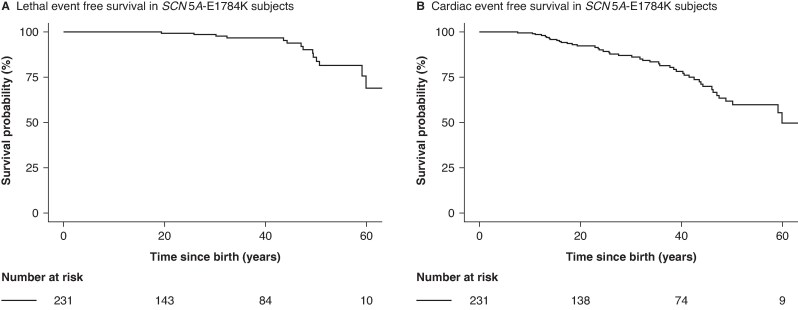
Lethal event (*A*) and cardiac event-free (*B*) survival from birth in *SCN5A*-E1784K subjects.

**Figure 3:**
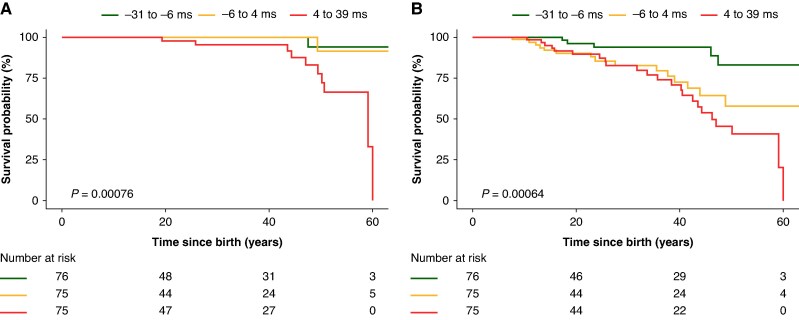
Lethal event (*A*) and cardiac event-free (*B*) survival in *SCN5A*-E1784K subjects classified based on residual QRS tertiles (i.e. difference between observed and age predicted QRS duration, ranges in ms).

Median follow-up was 25 years (IQR: 28). The lethal and cardiac event-free survival from birth in the total group is shown in *Figure 2*. The incidence of lethal or cardiac events per 100-person-years were 0.20 [95%CI: 0.19–0.20] and 0.67 [95%CI: 0.65–0.68], respectively. The observed survivals and incidence for lethal events and cardiac events for rQRS tertiles (i.e. age-corrected) are shown in *Figure 3* and *Table 3*. Incidence for lethal events was higher in the third rQRS tertile compared to the others. For cardiac events, the incidences increased with increasing rQRS tertile. Incidence rates per QRS category showed significant increasing rates with increasing QRS category, both for lethal and cardiac events (*Table 3*).

**Table 3:** Incidence of lethal and cardiac events per 100-person years per residual QRS duration (rQRS) tertiles and QRS <100, 100–120, and >120 ms [95% confidence interval]

rQRS	Lethal events	Cardiac events
[−31 to −6]	0.04 [0.04–0.04]	0.22 [0.21–0.23]
(−6 to −4]	0.05 [0.04–0.05]	0.71 [0.68–0.74]
(4 to 39]	0.44 [0.42–0.46]	1.10 [1.05–1.14]
QRS	Lethal events	Cardiac events
<100 ms	0.05 [0.05–0.05]	0.43 [0.43–0.45]
100–120 ms	0.30 [0.29–0.31]	0.84 [0.80–0.87]
>120 ms	0.57 [0.52–0.61]	1.73 [1.57–1.89]

In the long QT only subjects (*n* = 117), the incidence of lethal or cardiac events per 100-person-years were 0.13 [95%CI: 0.12–0.14] and 0.72 [95%CI: 0.69–0.75], respectively. The CCD only (*n* = 4) and BrS only (*n* = 14) groups were considered too small to calculate phenotype-specific event rates.

Event status and date data after diagnosis were only available for 97 *SCN5A*-E1784K subjects. Median follow-up was 2.5 years (IQR: 4). A total of four (9%) *SCN5A*-E1784K subjects experienced a lethal event after diagnosis. Nine (9%) cardiac events were observed during follow-up after the diagnosis. Event numbers and follow-up time were considered too small for sufficiently robust findings, but results for QRS, rQRS, PR, and rPR have been included as sensitivity analyses (see Supplementary material online, *Table S4*).

## Discussion

We are the first to report clinical characteristics associated with arrhythmic events in a large cohort with a single *SCN5A* variant: *SCN5A*-E1784K the commonest pathogenic *SCN5A* variant. A longer PR and rPR interval and higher QRS and rQRS duration were associated with increased occurrence of events in the overall cohort. Only rQRS was robustly associated with lethal and cardiac event-free survival, indicating ventricular conduction as a potential marker of risk.

### Cohort demographics

Approximately half of the cohort were Europeans, and a significant proportion were Japanese or of other/mixed ancestry, reflective of global prevalences. Although some clinical and ECG features were consistent, ancestry differences are apparent throughout the results such as use of provocation testing and antiarrhythmic medication. This is partly explained by variation in practice between centres internationally. The median age at the time of ECG acquisition was higher in patients with lethal and/or cardiac events. This probably reflects that older patients were more likely to have suffered a symptom than younger individuals given their lifetime exposure to risk.

Only a minority of cases received beta-blockers and the class 1b antiarrhythmic mexiletine, reflecting some of the challenges of pharmacotherapy as well as the latter’s utility for shortening the QTc and reducing events in LQT3.^9,10^

### Cardiac conduction and survival

QRS and PR were significantly greater in patients with lethal and cardiac events. This bears similarity to Meregalli *et al.*, who reported that BrS families with *SCN5A* variants with a more severe conduction phenotype had a greater likelihood of syncope^11^ and Tuijnenburg *et al.*, who reported a prolonged QRS interval in symptomatic *SCN5A* loss-of-function variant subjects.^12^ Similarly, Yamagata *et al.* found that BrS probands with *SCN5A* variants, especially those with greater biophysical effects, exhibited more cardiac conduction abnormalities and a higher risk of cardiac events.^13^

Thus, it is possible that the underlying mechanism of increased risk in *SCN5A*-E1784K patients is due to ventricular arrhythmia initiated by conduction slowing and re-entry. Indeed, 2 out of 12 patients with pacemakers experienced lethal events despite prevention of bradyarrhythmia. Our findings may also therefore be relevant to other *SCN5A* variants.

Interestingly, there was no association between LQTS phenotype and rQTc with lethal nor cardiac events. Although QT prolongation observed in *SCN5A*-E1784K subjects will most likely put them at higher risk compared to the general population, the variation in QTc observed among *SCN5A*-E1784K subjects is insufficient for further risk stratification within this group.^14^ Furthermore, there was no association of risk with Brugada phenotype, although this was limited by small numbers of patients with the spontaneous type 1 pattern or undergoing sodium channel blocker challenge.

### Clinical utility

The low overall annual incidence of lethal events (0.20%) and cardiac events (0.67%), most occurring at first presentation, indicates the pressing need to differentiate those at greatest risk of SCD for preventative therapy. However, the variation in clinical phenotype amongst E1784K-*SCN5A* subjects poses a challenge when determining optimal management strategies as illustrated by the variability in clinical management in the present cohort. Event-free survival (*Figure 3*) and the incidence rates per 100 person years permit some discrimination of risk using rQRS, albeit still crude. The lethal event incidence rates per 100 person years in the rQRS tertiles were 0.04, 0.05, and 0.44 for the first, second, and third tertile, respectively, while the cardiac event rates were 0.22, 0.71, and 1.10, for the first, second, and third tertile, respectively. This indicates a 10-fold higher risk of lethal events in the third tertile vs. the first and second and a three-fold to five-fold increase in cardiac event risk in the second and third tertiles compared to the first tertile. Thus, pending further validation of these exploratory findings, rQRS could guide preventative therapy for *SCN5A-*E1784K patients at lowest and greatest risk. Our findings also add to other data on loss of function *SCN5A* variants that may support a wider role for QRS as a risk marker.

Thus, by studying a large international cohort of *SCN5A-*E1784K patients, we were able to gain unique insights into the clinical spectrum and breadth of phenotypic variability of this variant. These findings have provided insights into risk stratification and may support more personalized approaches to medical management in these patients in the future. Additionally, this work may provide insights into other *SCN5A* disease, although further studies will be needed to confirm these findings.

### Limitations

This study was limited by retrospective data collection. Ancestry data were based on patient-reported ancestry and clinical records. There were some limitations in the definitions of disease phenotypes applied in this study, i.e. LQTS and CCD. However, in the context of the families studied, these were reasonable surrogates to use. The prevalence of BrS was underestimated because drug provocation testing had not been carried out in nearly two-thirds of the cohort reflective of variation in clinical practice globally^15^ and the lack of best practice guidelines for managing families with this unique variant.

Arrhythmic syncope was included in the cardiac event status (instead of focusing only on lethal events) due to the numbers available to increase statistical power. The diagnosis of arrhythmic syncope was specified by the recruiting cardiologist. While syncope may have been due to ventricular arrhythmias, we cannot completely exclude the possible inclusion of bradyarrhythmias or even atypical vasovagal syncope. Heart block and asystole were not recorded as cardiac events.

Follow-up data after diagnosis were limited and therefore event-free survival was analysed from birth. The present study does not allow estimation of the number of cardiac events occurring during follow-up and/or while the patient is on a beta-blocker or mexiletine because medication in general started after the first event and data on subsequent events were not systematically available. To mitigate for age-related variation in QRS, stratification of event-free survival utilized age-corrected rQRS values. Nonetheless, this approach cannot overcome all issues related to age-related changes in QRS duration.

Finally, the study is liable to immortality bias (i.e. subjects have to survive to the date of diagnosis to be included in the cohort), but effort was made to include as many *SCN5A*-E1784K subjects, and 70% of the events occurred within 2 years of the ECG date (range −40 to 20). Nevertheless, severely affected *SCN5A*-E1784K subjects might have been missed. In addition, due to the design of the study, estimates of medication effects are liable to disease severity bias (i.e. confounding by indication). However, only 2/45 cases were on medication prior to their event.

## Conclusion

Ventricular myocardial conduction, as evidenced by QRS duration, appears likely to play a role in the risk of arrhythmic events in patients with overlap sodium channel disease (*SCN5A*-E1784K). This demonstrates an important opportunity for the personalization of risk stratification in these patients and age-corrected QRS duration may potentially help identify patients who could benefit from preventative therapies.

## Supplementary Material

euag113_Supplementary_Data

## Data Availability

The data underlying this article will be shared on reasonable request to the corresponding author.
