# Supplementary material for: Ventricular conduction is a marker for arrhythmic risk in SCN5A-E1784K overlap sodium channel disease
Source: Europace. 2026 May 11;28(6):euag113. doi: 10.1093/europace/euag113 (PMC13237868; doi:10.1093/europace/euag113)
Supplement: euag113_Supplementary_Data [file euag113_supplementary_data.docx]

Supplemental materials

# Supplemental methods

# List of participating centers

- 1. Cardiovascular and Genomic Research Institute, City St George's University of London, London, United Kingdom; Cardiology Clinical Academic Group, St George's University Hospitals National Health Service (NHS) Foundation Trust, London, United Kingdom
  2. Queen Mary, University of London, London, United Kingdom
  3. University of Nottingham, Nottingham, United Kingdom
  4. Belfast Health and Social Care Trust, Belfast, United Kingdom; Queen’s University Belfast, Belfast, United Kingdom
  5. Dartford and Gravesham NHS Trust, Darent Valley Hospital, Dartford, United Kingdom
  6. Leeds Teaching Hospitals NHS Trust, Leeds, UK
  7. Mater University and Private Hospitals, Dublin, Ireland
  8. Amsterdam UMC, University of Amsterdam, Heart Center, Department of Clinical and Experimental Cardiology, Amsterdam Cardiovascular Sciences, Meibergdreef 9, Amsterdam, The Netherlands
  9. l’institut du thorax, INSERM, CNRS, UNIV Nantes, Nantes, France ; L’Institut du Thorax, Reference centre for hereditary arrhythmic diseases, Cardiologic department and U1087 Nantes, France; CHU Nantes, Service de Génétique Médicale, Nantes, France; l’institut du thorax, CHU Nantes, Service de Cardiologie, Nantes, France
  10. AP-HP, Hôpital Bichat, Département de Cardiologie et Centre de Référence des Maladies Cardiaques Héréditaires, Université Paris Diderot, Sorbonne Paris Cité, Paris, France INSERM U1166
  11. Istituto Auxologico Italiano, IRCCS, Center for Cardiac Arrhythmias of Genetic Origin, Milan, Italy; Istituto Auxologico Italiano, IRCCS, Laboratory of Cardiovascular Genetics, Milan, Italy; Istituto Auxologico Italiano, IRCCS, Department of Cardiovascular, Neural and Metabolic Sciences, San Luca Hospital, Milan, Italy; Department of Medicine and Surgery, University of Milano-Bicocca, Milan, Italy
  12. Rhythmology and Electrophysiology, Department of Cardiology and Angiology, Hannover Medical School, Hannover, Germany
  13. Department of Medicine, University Medical Centre Mannheim (UMM), Faculty of Medicine Mannheim, University of Heidelberg, European Center for AngioScience (ECAS), and DZHK (German Center for Cardiovascular Research) partner site Heidelberg/Mannheim, Mannheim, Germany
  14. Helmholtz Zentrum München, Institute of Human Genetics, Neuherberg, Germany; Technische Universität München, Institute of Human Genetics, Munich, Germany; DZHK (German Centre for Cardiovascular Research), Partner Site Munich Heart Alliance, Munich, Germany
  15. Departments of Cardiovascular Medicine (Division of Heart Rhythm Services), Pediatric and Adolescent Medicine (Division of Pediatric Cardiology), and Molecular Pharmacology & Experimental Therapeutics (Windland Smith Rice Sudden Death Genomics Laboratory), Mayo Clinic, USA
  16. Vanderbilt University School of Medicine, Nashville, USA
  17. Shiga University of Medical Science, Shiga, Japan
  18. National Cerebral and Cardiovascular Center, Osaka, Japan
  19. Kizawa memorial hospital, Gifu, Japan
  20. Nippon Medical School, Tokyo, Japan

# Statistical analysis

## Additional statistical analyses

For the logistic regression models, the linearity assumption of continuous variables was checked by comparing the fit of a model assuming a nonlinear relationship (restricted cubic splines with 3 knots) to that of a model assuming a linear relationship using a likelihood ratio test. In addition, a visual inspection was done by plotting the logit values against the continuous variable.

Possible ancestry specific risk factors were investigated by comparing the fit of a model with the main effects only with that of a model with main and the interaction effect using a likelihood ratio test. In case of significant interaction, odds ratios were estimated in separate ancestries.

## Additional sensitivity analyses

### Removal of the three largest families

To examine the influence of the three largest families (> 10 members), analyses were repeated with exclusion of these families. The resulting sample size was 163.

### Long QT individuals only

Although no significant difference in the effect of residual QRS duration on (cardiac or lethal) events was found between long QT or BrS patients, analyses were repeated in the 109 patients with Long QT. Too few patients were available for the other phenotypes to be analysed separately.

### Without SCN5A-E1784K subjects with no (clear) phenotype

To examine the possible effect of including asymptomatic SCN5A-E1784K individuals in the analysis.

### Starting follow up at date of the ECG recording

To examine the possible immortality and look ahead bias, survival analyses were repeated with follow up starting at the day of the ECG recording instead at birth.

# Results piecewise linear regression

Relationship ECG parameters and age at ECG.

The results of the piecewise linear modelling were:

$$\hat{PR interval} (ms)=f(Age at ECG)\left\{ \begin{aligned} 119.4+3.1 \times Age at ECG, Age at ECG<13 \\ 152.0+0.6 \times Age at ECG, Age at ECG\geq13 \end{aligned} \right.$$

$$\hat{QRS duration (ms)}=f\left( Age at ECG \right)\left\{ \begin{aligned} 80.6+1.2 \times Age at ECG, Age at ECG<14 \\ 96.4+0.1 \times Age at ECG, Age at ECG\geq14 \end{aligned} \right.$$

$$\hat{QTc interval} (ms)=f\left( Age at ECG \right)\left\{ \begin{aligned} 441.8+8.4 \times Age at ECG, Age at ECG<7 \\ 503.3-0.4 \times Age at ECG, Age at ECG\geq7 \end{aligned} \right.$$

$$\hat{RR interval} (s)=f(Age at ECG)\left\{ \begin{aligned} 0.403+0.072 \times Age at ECG, Age at ECG<7 \\ 0.900+0.002\times Age at ECG, Age at ECG\geq7 \end{aligned} \right.$$

# Supplemental Tables

Table S1: Clinical and ECG characteristics of SCN5A-E1784K and their relatives at the time of the ECG recording. Differences between ancestries were analyzed with the Fisher exact or Kruskal-Wallis test and statistically significant values (p<0.05) are highlighted.

|  | Total cohort  (n=335) | European  (n=175) | Japanese  (n=80) | Other/mixed ancestry  (n=80) | p-value |
| --- | --- | --- | --- | --- | --- |
| Male | 148 (44%) | 72 (42%) | 41 (51%) | 35 (44%) | 0.311 |
| Age at baseline ECG (years) | 28.6 (29.3) | 35.3 (29.2) | 17.6 (29.7) | 29.9 (22.7) | 0.006 |
| *SCN5A*-E1784K | 231 (69%) | 126 (72%) | 65 (81%) | 40 (50%) | <0.001 |
| Medication | 60 (18%) | 44 (25%) | 9 (11%) | 7 (9%) | 0.001 |
| BrS phenotype | 70 (21%) | 47 (27%) | 6 (8%) | 17 (21%) | 0.002 |
| ECG parameters* |  |  |  |  |  |
| PR interval (ms) | 161 ± 28 | 158 ± 30 | 164 ± 28 | 164 ± 25 | 0.022 |
| QRS duration (ms) | 94 ± 13 | 95 ± 14 | 95 ± 12 | 90 ± 14 | 0.004 |
| QRS axis (degrees) | 56 (50) | 53 (50) | 68 (39) | 52 (49) | 0.014 |
| Abnormal QRS axis | 47 (14%) | 21 (12%) | 13 (16%) | 13 (16%) | 0.556 |
| QTc interval (ms) | 468 ± 42 | 461 ± 35 | 497 ± 41 | 454 ± 43 | <0.001 |
| Long QT (QTc > ms) | 193 (59%) | 93 (54%) | 66 (83%) | 34 (43%) | <0.001 |
| RR interval (s) | 0.90 ± 0.20 | 0.87 ± 0.19 | 0.98 ± 0.20 | 0.86 ± 0.18 | <0.001 |
| Data are presented as count (%), mean ± SD or median (interquartile range)  *Available for n=330, unless indicated otherwise | | | | | |

Table S2: Breakpoint, intercept and slope of the piecewise linear regression models. These models were used to calculate the expected value for a given age. Residuals were subsequently calculated by subtracting the observed value from the expected value.

| PR interval (ms), break point age at ECG 13 years (3 - 8, p<0.001) | | | |
| --- | --- | --- | --- |
| Age at ECG | Intercept | Slope (95% CI) | P-value |
| <13 years | 119.4 | 3.1 (1.3 - 4.9) | <0.001 |
| ≥13 years | 152.0 | 0.6 (0.3 - 0.8) | <0.001 |
|  |  |  |  |
| QRS duration (ms), break point age at ECG 14 years (8 - 20, p<0.001) | | | |
| Age at ECG | Intercept | Slope (95% CI) | P-value |
| <14 years | 80.1 | 1.2 (0.4 - 1.9) | 0.003 |
| ≥14 years | 96.4 | 0.1 (-0.1 - 0.2) | 0.32 |
|  |  |  |  |
| QTc (ms), break point at ECG 7 years (5 − 9, p<0.001) | | | |
| Age at ECG | Intercept | Slope (95% CI) | P-value |
| <7 years | 441.8 | 8.4 (3.4 - 13.3) | 0.001 |
| ≥7 years | 503.3 | -0.4 (-0.6 - -0.2) | 0.001 |
|  |  |  |  |
| RR interval (s), break point age at ECG 7 years (5 - 9, p<0.001) | | | |
| Age at ECG | Intercept | Slope (95% CI) | P-value |
| <7 years | 0.403 | 0.072 (0.004 - 0.105) | <0.001 |
| ≥7 years | 0.900 | 0.002 (0.000 - 0.003) | 0.047 |

Table S3: ECG characteristics for female and male *SCN5A*-E1784K subjects

|  | Females | Males | P-value |
| --- | --- | --- | --- |
|  | (n=121) | (n=110) |  |
| Age at ECG (years) | 34 (29) | 22 (31) | 0.01 |
| PR interval (ms) | 163 ± 26 | 167 ± 33 | 0.30 |
| QRS duration (ms) | 95 ± 11 | 98 ± 15 | 0.04 |
| QTc interval (ms) | 491 ± 31 | 488 ± 30 | 0.50 |
| LQTS* | 92 (77%) | 96 (90%) | 0.02 |
| RR interval (ms) | 0.92 ± 0.20 | 0.92 ± 0.22 | 0.90 |
| BrS phenotype | 37 (31%) | 29 (26%) | 0.57 |

Data are presented as count (%), mean ± SD or median (interquartile range). * LQTS was defined as a QTc >470ms for females and >450ms for males

|  | Lethal events | | Cardiac events | |
| --- | --- | --- | --- | --- |
| Change from original analysis | OR [95% CI] | P-value | OR [95% CI] | P-value |
| Largest three families removed (n = 167) | 6 lethal events | | 34 cardiac events | |
| PR interval (ms) | 1.04 [1.00 - 1.08] | 0.068 | 1.02 [1.00 - 1.03] | 0.019 |
| Residual PR (ms) | 1.03 [0.98 - 1.07] | 0.270 | 1.01 [0.99 - 1.03] | 0.302 |
| QRS duration (ms) | 1.06 [1.02 - 1.10] | 0.004 | 1.04 [1.02 - 1.07] | 0.001 |
| Residual QRS (ms) | 1.05 [1.01 - 1.10] | 0.022 | 1.03 [1.01 - 1.06] | 0.007 |
|  |  |  |  |  |
| Long QT only (n=117) | 4 lethal events | | 21 cardiac events | |
| PR interval (ms) | 1.00 [0.97 - 1.03] | 0.960 | 1.02 [1.00 - 1.04] | 0.041 |
| Residual PR (ms) | 0.99 [0.95 - 1.03] | 0.524 | 1.01 [0.98 - 1.03] | 0.549 |
| QRS duration (ms) | 1.14 [1.09 - 1.19] | <0.001 | 1.08 [1.04 - 1.13] | <0.001 |
| Residual QRS (ms) | 1.15 [1.06 - 1.25] | 0.001 | 1.07 [1.03 - 1.11] | <0.001 |
|  |  |  |  |  |
| Individuals without phenotype removed (n=205) | 12 lethal events |  | 41 cardiac events |  |
| PR interval (ms) | 1.04 [1.01 - 1.06] | 0.012 | 1.02 [1.01 - 1.03] | 0.002 |
| Residual PR (ms) | 1.03 [1.01 - 1.06] | 0.017 | 1.01 [1.00 - 1.03] | 0.081 |
| QRS duration (ms) | 1.09 [1.09 - 1.12] | <0.001 | 1.07 [1.04 - 1.10] | <0.001 |
| Residual QRS (ms) | 1.08 [1.08 - 1.12] | <0.001 | 1.06 [1.03 - 1.09] | <0.001 |
|  |  |  |  |  |
|  | Lethal events | | Cardiac events | |
| Change from original analysis | HR [95% CI] | P-value | HR [95% CI] | P-value |
| Survival analysis, follow-up starting at date of diagnosis (n=97) | 4 lethal events |  | 9 cardiac events |  |
| PR interval (ms) | 1.03 [0.97 - 1.09] | 0.290 | 1.02 [1.00 - 1.04] | 0.019 |
| Residual PR (ms) | 1.03 [0.96 - 1.11] | 0.360 | 1.03 [1.00 - 1.05] | 0.037 |
| QRS duration (ms) | 1.07 [0.76 - 1.50] | 0.700 | 1.04 [1.00 - 1.09] | 0.071 |
| Residual QRS (ms) | 1.06 [0.93 - 1.22] | 0.390 | 1.04 [0.98 - 1.09] | 0.194 |
| OR: odds ratio; HR: hazard ratio; 95% CI: 95% confidence interval; | | | | |

Table S4: Sensitivity analyses: odds and hazard ratios estimated for (residual) PR interval and (residual) QRS in SCN5A-E1784K using logistic / Cox proportional hazard regression with adjustment for relatedness.

# Supplemental figure

Figure S1: Relationship between age and A) PR interval, B) QRS duration, C) QTc and 4) RR interval in *SCN5A-*E1784K subjects.


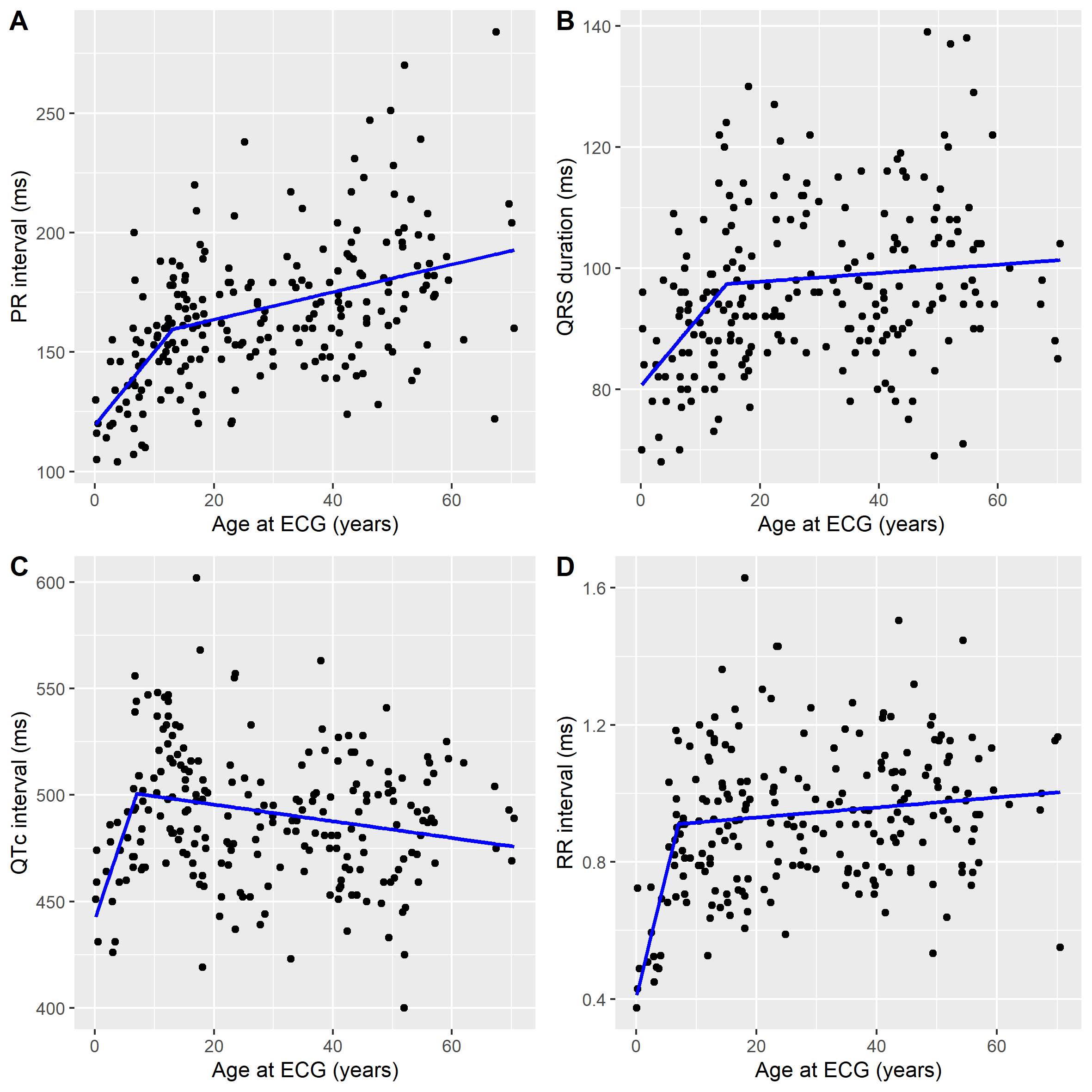


Figure S1
